# Angioimmunoblastic T cell lymphoma: novel molecular insights by mutation profiling

**DOI:** 10.18632/oncotarget.14846

**Published:** 2017-01-27

**Authors:** Ming Wang, Shaowei Zhang, Shih-Sung Chuang, Margaret Ashton-Key, Eguzkine Ochoa, Niccolo Bolli, George Vassiliou, Zifen Gao, Ming-Qing Du

**Affiliations:** ^1^ Division of Molecular Histopathology, Department of Pathology, University of Cambridge, Cambridge, UK; ^2^ Department of Pathology, Health Science Centre, Peking University, Beijing, China; ^3^ Department of Pathology, Chi-Mei Medical Center, Tainan, Taiwan; ^4^ Department of Cellular Pathology, Southampton University Hospitals National Health Service Foundation Trust, Southampton, UK; ^5^ Wellcome Trust Sanger Institute, Wellcome Trust Genome Campus, Hinxton, Cambridge, UK; ^6^ Department of Oncology and Onco-Hematology, University of Milan, Milan, Italy; ^7^ Department of Hematology and Pediatric Onco-Hematology, Fondazione IRCCS Istituto Nazionale dei Tumori, Milan, Italy

**Keywords:** AITL, WES, somatic mutation, oncogenic mechanism

## Abstract

Angioimmunoblastic T cell lymphoma (AITL) originates from follicular helper T-cells and is characterised by a polymorphic infiltrate with the neoplastic T-cells forming small clusters around the follicle and high endothelial venules. Despite the recent advances in its phenotypic characterisation, the genetics and molecular mechanisms underlying AITL are not fully understood. In the present study, we performed whole exome sequencing in 9 cases of AITL from Taiwan (*n* = 6) and U.K. (*n* = 3). We confirmed frequent mutations in *TET2* (9/9), *DNMT3A* (3/9), *IDH2* (3/9), *RHOA* (3/9) and *PLCG1* (2/9) as recently reported by others. More importantly, we identified mutations in *TNFRSF21* (1/9), *CCND3* (1/9) and *SAMSN1* (1/9), which are not yet seen or strongly implicated in the pathogenesis of AITL. Among the pathogenic mutations identified in AITL, mutations in DNA methylation regulators *TET2* and *DNMT3A* occur early in hematopoietic stem cells as shown by previous studies, and these genetic events enhance the self-renewal of hematopoietic stem cells, but are unlikely to have any major impact on T-cell differentiation. Mutations in *RHOA*, *PLCG1* and *TNFRSF21* (DR6), which encode proteins critical for T-cell biology, most likely promote T-cell differentiation and malignant transformation, consequently generating the malignant phenotype. Our findings extend the molecular insights into the multistage development of AITL.

## INTRODUCTION

Angioimmunoblastic T-cell lymphoma (AITL) is a common subtype of peripheral T-cell lymphoma (PTCL). The vast majority of AITL are associated with EBV infection, however, the neoplastic T-cells are EBV negative. Histologically, AITL is characterized by a polymorphic infiltrate with the neoplastic T-cells typically expanding within a background of prominent arborizing high endothelial venules and perivascular proliferation of follicular dendritic cells. The polymorphic infiltrate comprises small reactive lymphocytes, B-immunoblasts (often positive for EBV), plasma cells, eosinophils and macrophages.

AITL originates from follicular helper T-cells and the neoplastic T-cells express all the cardinal features of follicular helper T-cells, including CXCR5,CXCL13, ICOS, PD-1, BCL6, SAP and c-MAF, and preserve at least the major function of follicular helper T-cells, for example, help for antibody production by B-cells [1, 2]. A high-affinity TCR is a characteristic feature of follicular helper T-cells, and is essential for the commitment of CD4+ T cells to differentiate into follicular helper T-cells and also critical for their maintenance and survival. In view of these, TCR signaling may play a critical role in the pathogenesis of AITL.

The genetics of AITL is beginning to unfold. t(5;9) (q33;q22)/ITK-SYK, originally identified in the follicular variant of PTCL, has been also found in AITL albeit rather infrequent [3–5]. The ITK-SYK fusion product associates constitutively with lipid rafts in T-cells and triggers antigen-independent phosphorylation of TCR-proximal proteins and activation of their downstream pathways [6, 7]. More recent studies by whole exome sequencing (WES) and targeted sequencing have identified a wide spectrum of somatic mutations in AITL and among those, mutations in *TET2*, *DNMT3A*, *IDH2, RHOA* and CD28 are the most frequent [8–15]. Nonetheless, the number of AITL cases investigated by whole exome sequencing in each of the above studies is small (≤ 5) and the full spectrum of the mutation profile of AITL most likely remains incompletely characterized. In this study, we report the exome mutation profile of 9 well-characterized AITL with emphasis on the novel mutations identified.

## RESULTS AND DISCUSSION

### Overview of discoveries by WES

WES generated an average of 25.7 million reads per sample, with an average of 89% of the target sequences being covered by > 10 reads (Supplementary Table 1). As a sufficient amount of matched germline DNA for WES was available only in one case, we performed extensive data filtering to remove the known SNPs reported in databases including the 1000 genome project that contains variants from various ethnic populations including Han Chinese. We also removed the variants that were predicted to lack functional impact by 3 of the 7 mutation functional prediction programs. Consequently, a total of 603 variants in 527 genes were detected in 9 AITL (average 67/case; range 10–90/case) (Supplementary Figure 1). Nonetheless, the number of variants in cases 2–9 was most likely overestimated due to a lack of corresponding germline DNA for WES.

**Figure 1:**
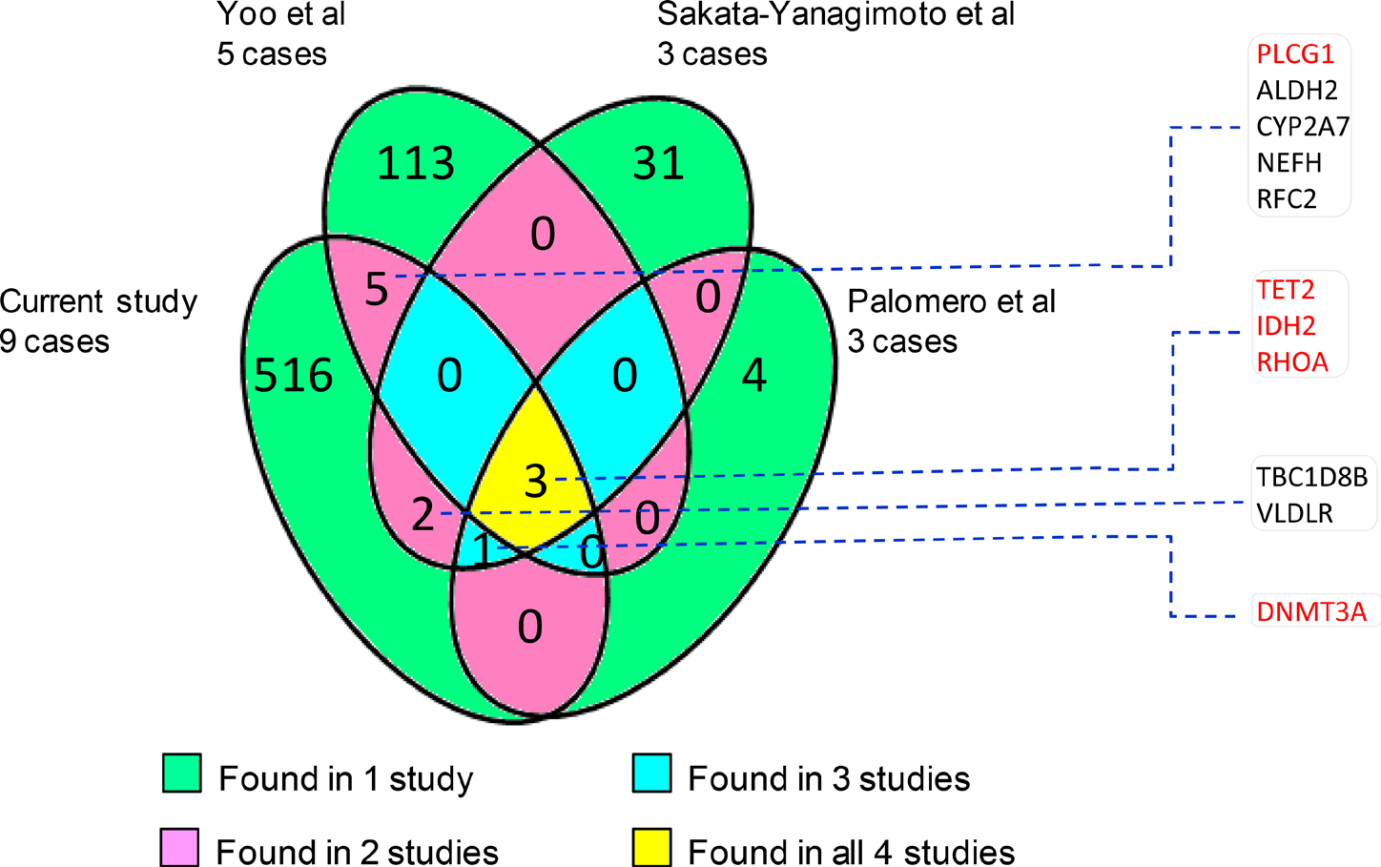
Number of common and unique genes that are found to harbour mutations by different whole exome sequencing studies [8–10] In general, there is little overlap among the mutated genes identified by the 4 different studies. The 11 overlap genes with other studies are listed and those known pathogenic are highlighted in red.

To examine whether this study identified any novel mutations, we compared our data with those of the recent studies by WES and targeted sequencing [8–11]. To our surprise, there was little overlap in the mutation landscape among these different studies despite finding of mutations in a small common set of genes (Figure 1), suggesting genetic heterogeneity in AITL and incomplete mutation discoveries in each of these studies. In addition, the small number of cases investigated, variability in tumor cell content, sequence coverage and efficacy of variant calling most likely contributed to the discrepancy among these different studies. Among a total of 527 mutated genes identified in this study, only 11 were found to be mutated in AITL by previous WES studies.

### Pathogenic mutations identified by WES

**Figure 2:**
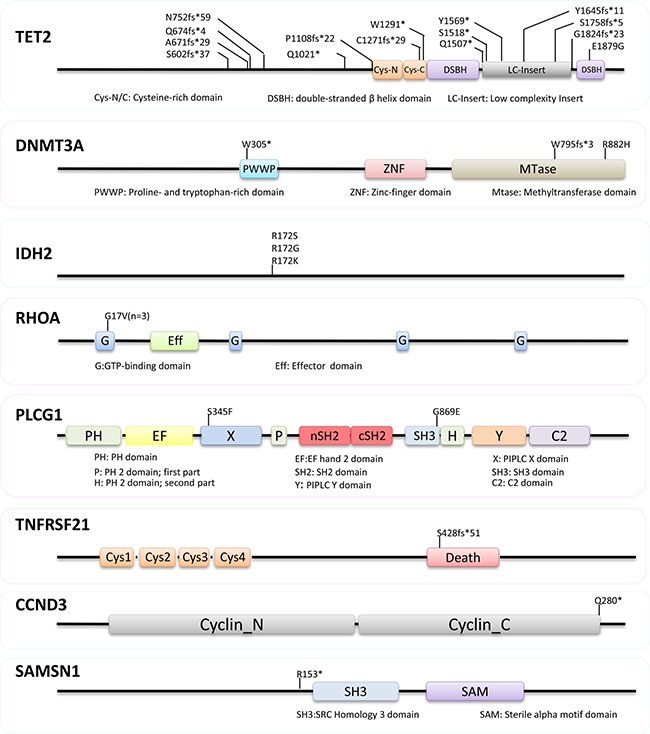
Nature and distribution of mutations in *TET2, DNMT3A, IDH2, RHOA, PLCG1, TNFRSF21, CCND3* and *SAMSN1* The vast majority (14/15 = 93%) of *TET2* mutations were frameshift or nonsense changes without any clusters. *DNMT3A* mutation was found in three cases, with 2 of the 3 mutations being frameshift or nonsense changes. Mutation in *IDH2, RHOA* and *PLCG1* are exclusively missense changes, while those in *TNFRSF21, CCND3* and *SAMSN1* are deleterious alterations.

**Figure 3:**
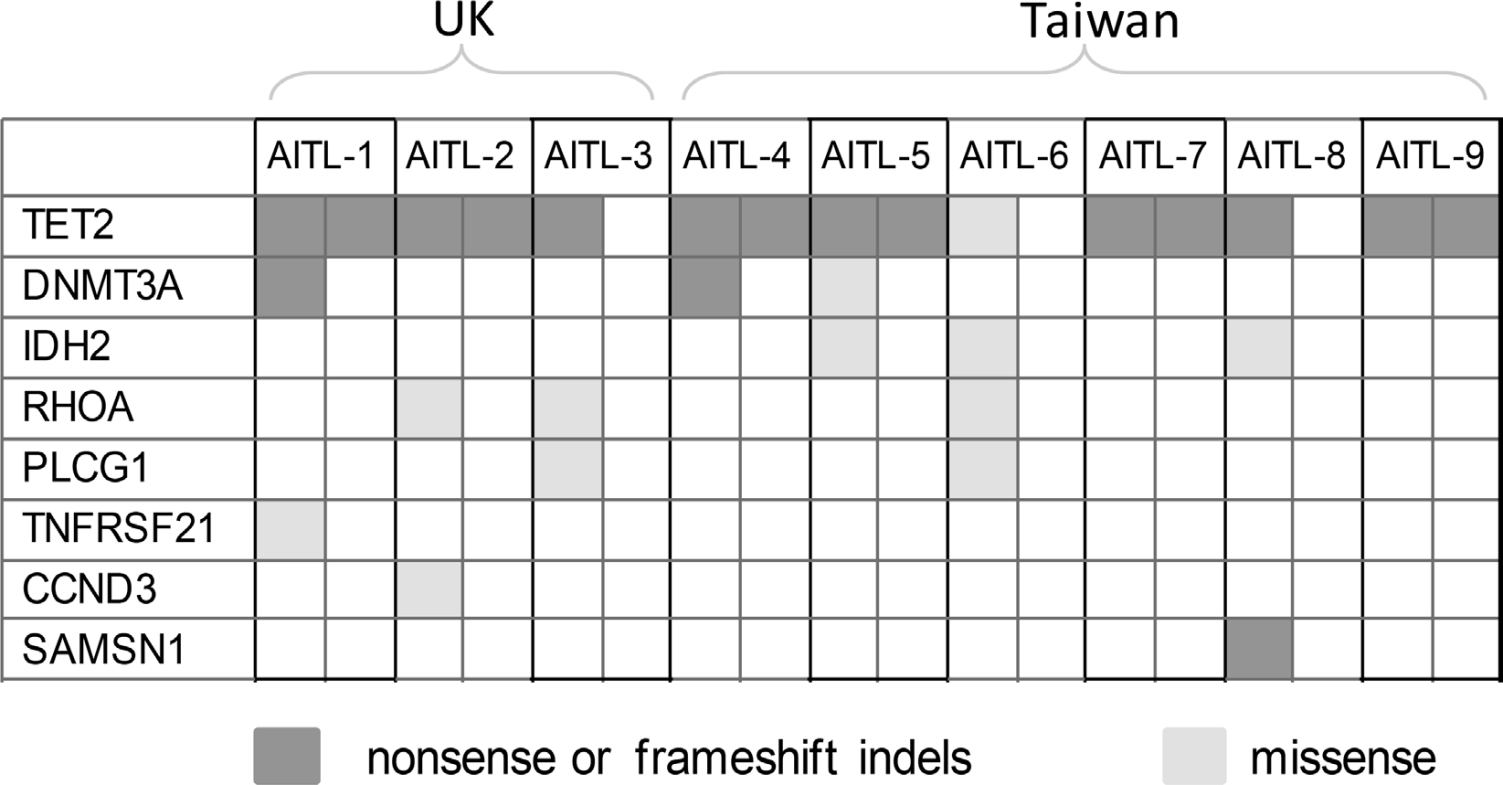
Distribution of the shortlisted pathogenic mutations in 9 cases of AITL investigated by WES There is no apparent association among the mutations identified.

We confirmed frequent mutations of *TET2* (9/9 cases), *DNMT3A* (3/9), *IDH2* (3/9 cases) and *RHOA* (3/9 cases) in AITL (Figures 2 and 3, Supplementary Table 2), which were identified by previous WES and targeted sequencing [8–11]. In line with previous studies, *TET2* mutations were frequently multiple, with 2 mutations seen in 6 cases. The vast majority (14/15 = 93%) of *TET2* mutations were frameshift or nonsense changes, and rather widely distributed without any clusters, suggesting that these mutations were inactivating events. *DNMT3A* mutation was found in three cases, with 2 of the 3 mutations being frameshift or nonsense changes. *DNMT3A* mutation was found exclusively in cases with double *TET2* mutations. *IDH2* mutation affected the same codon although resulting in different substitutions in each of the 3 cases involved, while *RHOA* mutation was exclusively G17V change in all 3 cases involved.

In addition to the above well-established mutations in AITL, we also identified mutations in several genes not yet seen or strongly implicated in the pathogenesis of AITL and they included *PLCG1*, *TNFRSF21*, *CCND3* and *SAMSN1* (Figures 2 and 3, Supplementary Table 2). All the variants identified by WES and included in the Supplementary Table 2 were confirmed by Sanger sequencing.

*PLCG1* was mutated in 2 of the 9 cases of AITL investigated by WES, and both mutations were missense changes, with *PLCG1* S345F reported previously in cutaneous T-cell lymphoma and nodal peripheral T-cell lymphoma [16, 17], and *PLCG1* G869E as a novel change. *PLCG1* S345F, localized in the N-terminal part of the “split” PH domain, was an activating change, enhancing the ability of PLCγ1 to activate NFAT, a transcriptional factor critical for T-cell activation and function [16]. *PLCG1* G869E is in the SH3 domain of the regulatory region, which along with the CSH2 domain, forms an interface with the CSH2-SH3 linker, serving as an auto-inhibitory interaction [18, 19]. *PLCG1* G869E, like R707Q in the CSH2 domain, may cause conformational changes, abolish such auto-inhibitory interaction, and thus activate PLCγ1 [20]. While revision of our manuscript, recurrent *PLCG1* mutations including S345F and G869E have also been reported in AITL by Vallois and colleagues, and most *PLCG1* mutations including S345F and G869E are gain of functional changes as shown by their ability to activate MALT1 and NFAT *in vitro* [21]. PLCγ1 plays an essential role downstream of TCR signaling by hydrolyzing phosphatidylinositol 4,5-bisphosphate to form inositol 1,4,5-triphosphate and diacylglycerol. *PLCG1* mutation may cause its constitutive activation and augment TCR signalling, thereby contributing AITL development.

*TNFRSF21*, encoding the death receptor-6 (DR6), was mutated in 1 of the 9 AITLs investigated by WES. The mutation is a frameshift deletion in the death domain, and most likely leads to inactivation of its encoded protein product. DR6 appears to act as a negative regulator to dampen immunoreceptor-coupled signaling pathways [22, 23], although its molecular function is still elusive. In absence of DR6, CD4+ T cells are hyper-proliferative and show a profound polarization toward a Th2 phenotype in response to TCR stimulation [22, 24, 25]. In light of these findings, *TNFRSF21* inactivation is an attractive pathogenic mechanism in AITL development. In addition to genetic changes, it is pertinent to investigate other potential mechanisms such as epigenetic changes that may inactivate *TNFRSF21* in AITL in future studies.

*CCND3* was mutated in 1 of the 9 AITLs investigated by WES, and the mutation generated a premature stop codon, predicting a truncated protein product. Similar nonsense or frameshift mutations have been recently reported in Burkitt lymphoma [26, 27]. These mutations eliminate the cyclin D3 carboxyl terminus that contains a phosphorylation motif, highly conserved among cyclin D3 homologs. This phosphorylation motif is critical for phosphorylation and polyubiquitination of D-type cyclin and thus their proteosome degradation [28]. As expected, these mutations markedly increase cyclin D3 stability and enhance its ability in driving cell proliferation [27].

*SAMSN1* was mutated in 1 of the 9 AITLs investigated by WES, and the mutation generated a stop codon upstream of the SH3 domain, thus most likely being a loss of function change. *SAMSN1* is a tumour suppressor gene in multiple myeloma. Germline deletion of *Samsn1* in mice predisposes to monoclonal gammopathy of undetermined significance and multiple myeloma, while in human *SAMSN1* expression is inactivated by promoter methylation [29]. In addition, SAMSN1 is a negative regulator of cell proliferation as shown by *in vitro* studies [30]. Thus, SAMSN1 mutation seen in AITL is highly likely pathogenic.

### Evidence for stepwise acquisition of genetic abnormalities with distinct oncogenic properties

**Figure 4:**
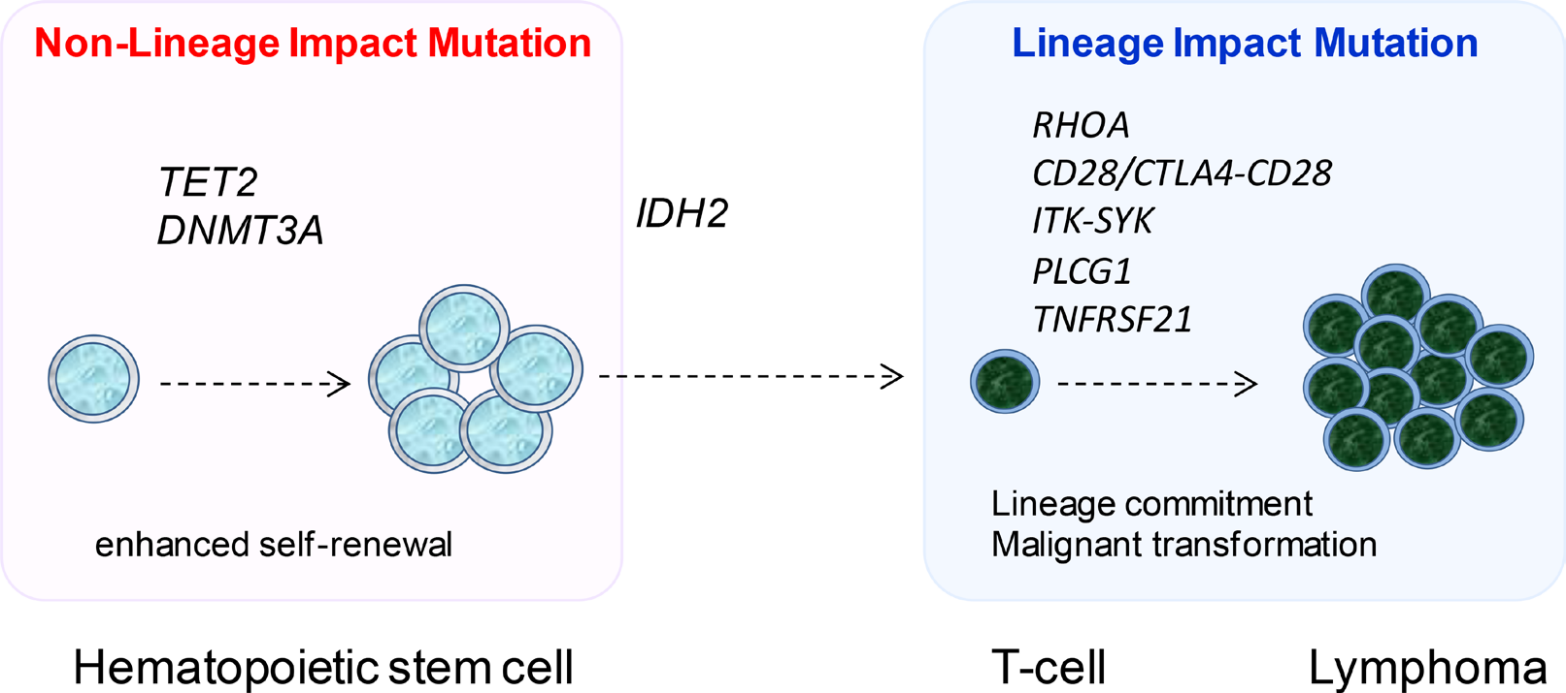
The proposed model of multistage development in AITL Mutations in DNA methylation regulators namely *TET2*, *DNMT3A* and *IDH2* are early events, with *TET2* and *DNMT3A* mutation occurring in hematopoietic stem cells. These genetic events enhance the self-renewal of hematopoietic stem cells, and do not have any major impact on cell lineage specific differentiation, thus are found in a variety of haematological malignancies. Mutation in *RHOA*, *PLCG1*, *TNFRSF21, CD28* and the *CTLA4-CD28 and ITK-SYK* fusion occurs late, and these genetic events affect proteins critical to T-cell biology, thus most likely promote T-cell differentiation as well as malignant transformation, consequently generating the malignant phenotype.

Despite the diverse spectrum of somatic mutations identified in AITL by whole exome and targeted sequencing, there is a small set of genes including *TET2*, *DNMT3A, IDH2* and *RHOA*, which are commonly found to be mutated by different studies including the present study [8–11]. Among these, *TET2*, *DNMT3A* and *IDH2* mutations are also found in a range of hematological malignancies with *IDH2* mutation additionally seen in several types of solid tumor, particularly low grade gliomas [31, 32]. Interestingly, *IDH2* mutation in AITL, like glioma, exclusively affects the R172 residue [8, 11, 12, 15, 32], but involves both R140 and R172 residues in acute myeloid leukemia and myelodysplastic syndromes with R140 mutation being more frequent [33, 34]. *TET2* and *DNMT3A* mutation appear to occur at an early stage of hematopoietic cell differentiation as these mutations are also found in non-malignant hematopoietic cells of patients with PTCL as well as normal elderly individuals [9, 35–37]. Their involvement in various malignancies and occurrence in hematopoietic progenitor cells are in keeping with their general role in regulation of DNA methylation, and the observation of an expanded hematopoietic stem cell pool in mice when *Tet2* or *Dnmt3a* is deleted or *Idh2* mutant is expressed in mouse bone marrow cells [38, 39]. Taken together, these findings suggest that *TET2*, *DNMT3A* and *IDH2* mutations are unlikely to have a major role in driving cell differentiation towards to the T-cell lineage, thus require cooperating events, which are crucial for the development and biology of T-cells, in the genesis of AITL (Figure 4).

The other frequently mutated gene found by different studies is *RHOA*, which is a small GTPase and alternates between an active GTP-bound configuration and an inactive GDP-bound state. The RHOA Gly17Val mutant does not bind GTP and inhibits the wild-type RHOA function by sequestering the RHOA guanine nucleotide exchange factors [8, 9]. RhoA has been shown to play important roles in the development and differentiation of thymocytes as well as in the biology of mature T-cells including TH2 cell differentiation [40, 41]. Although the precise molecular mechanisms of RHOA in T-cell biology remain to be investigated, it is pertinent to speculate that RHOA mutation may occur later than those in the aforementioned DNA methylation regulators, and its functional deregulation by mutation may promote T-cell differentiation and malignant transformation (Figure 4). In line with this notion, a recent study by RNA sequencing has identified a novel *CTLA4-CD28* fusion in AITL and other T-cell lymphoma subtypes [42]. The fusion combines the extracellular domain of CTLA4 with the cytoplasmic region of CD28, thus converting inhibitory signals into stimulatory signals for T-cell activation [42]. Interestingly, there is a considerable overlap between *CTLA4-CD28* fusion and *RHOA* mutation in AITL [42], suggesting possible oncogenic cooperation between the two events. In addition, CD28 is frequently activated by mutation in AITL with the D124 mutant showing increased affinity for ligand CD86 and the T195 mutant displaying increased affinity for intracellular adaptor proteins GRB2 and GADS/GRAP2 [14]. In view the established role of RHOA and CD28 in T-cell biology, *RHOA* mutation and *CTLA4-CD28* fusion/CD28 mutation could be regarded as T-cell lineage impact events, i.e. determining the phenotype of transformed cells. In this context, the present study identifies further genetic changes, namely *PLCG1* and *TNFRSF21* mutation, of this category in AITL (Figure 4).

## MATERIALS AND METHODS

### Patients samples

High molecular weight (HMW) DNA samples were extracted from fresh frozen specimens of 9 AITL from Department of Pathology, Chi-Mei Foundation Hospital, Taiwan (*n* = 6), and Department of Cellular Pathology, Southampton University Hospitals National Health Service Trust, Southampton, U.K. (*n* = 3) (Supplementary Table 3). The lymphoma diagnosis was established according to the 2008 WHO classification of tumours of haematopoietic and lymphoid tissues. In 6 cases where indicated, germline DNA were extracted from non-neoplastic formalin-fixed paraffin-embedded (FFPE) tissues or peripheral blood samples not involved by lymphoma, but sufficient amount of DNA was only available in 1 case for WES (Supplementary Table 3). Local ethical guidelines were followed for the use of archival tissues for research with the approval of the ethics committees of the involved institutions.

### Whole exome sequencing and somatic variant calling

These were carried out by the Wellcome Trust Sanger Institute. WES was successfully performed on 9 AITL DNA samples and 1 matched germline DNA sample as described previously [43]. Genomic libraries were prepared using the Illumina Paired End Sample Prep Kit, and target enrichment was performed using the Agilent SureSelect Human All Exon 50Mb kit. Each exome was sequenced using a 75bp paired-end protocol on an Illumina HiSeq platform. Sequencing reads were aligned to the hg19 reference genome using the BWA algorithm on default settings.

Variants were called using the Caveman and Pindel algorithms and subjected to a series of post processing filters to remove SNPs presented in germline DNA from this study, known SNPs ( ≥ 1%) in databases and in both east Asian and European populations of the 1000 genomes project. In addition, the variants discovered were checked against the COSMIC database to identify potential somatic and pathogenic changes. Finally, the variants discovered were further analyzed with 7 mutation functional prediction programs including SIFT, Polyphen-2 HDIV, Polyphen-2 DVAR, Mutation Assessor, FATHMM, GERP++ and SiPhy-29, and those predicted to be damaging by 5 or more programs were included in the final variant list.

### Somatic variant validation by PCR and sanger sequencing

Where indicated, novel variants identified by WES were confirmed by PCR and Sanger sequencing using the BigDye Terminator 3.1 System (Applied Biosystems, UK) on an ABI 3730 instrument (Applied Biosystems, UK), and their somatic nature was ascertained by analysis of germline DNA.

## SUPPLEMENTARY MATERIALS FIGURE AND TABLES


