# Supplementary material for: Angioimmunoblastic T cell lymphoma: novel molecular insights by mutation profiling
Source: Oncotarget. 2017 Jan 27;8(11):17763–70. doi: 10.18632/oncotarget.14846 (PMC5392284; doi:10.18632/oncotarget.14846)
Supplement: Supplementary file 1 [file oncotarget-08-17763-s001.pdf]

Angioimmunoblastic T cell lymphoma: novel molecular insights by mutation profiling

Supplementary Materials

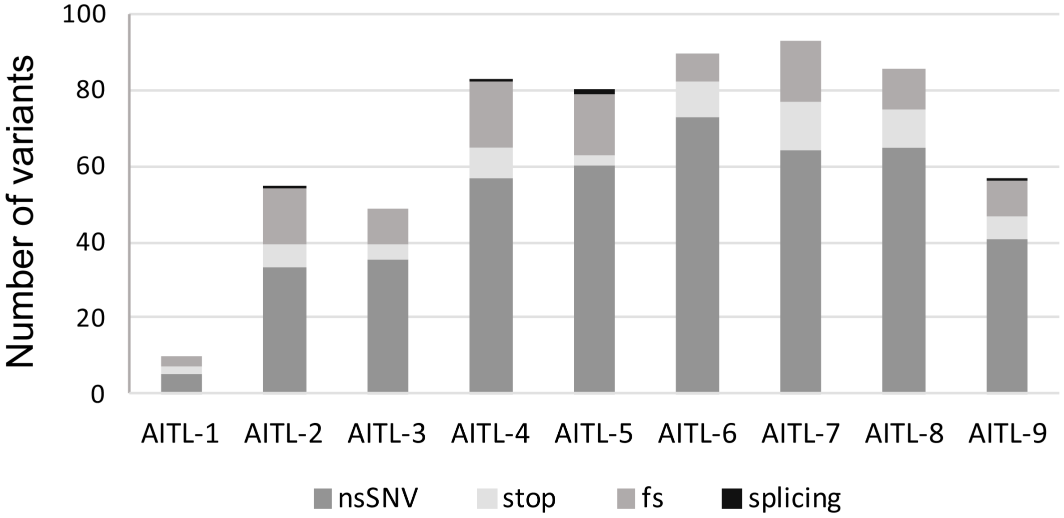

Supplementary Figure 1: Overview of the total number of putative pathogenic variants identified in AITL by whole exome sequencing. nsSNV: non synonymous single nucleotide variants; stop: nonsense change; fs: frameshift insertion or deletion; splicing: variants affecting the essential splicing site.

Supplementary Table 1: WES performance data

Sample ID	Nature of specimen used for WES	Total Reads (PE)	Total Bases (Gb)	Unmapped reads(%)	Mapped (Gb)	Mapped Reads (%)	Unique mapped (Gb)	Unique mapped Reads (%)	Coverage > 10 reads (%)	Coverage > 30 reads (%)	% on target (UMR)
AITL1_N	FFPE-T	24719677	3.708	2.7	3.585	96.7	3.165	88.3	89.6	65.1	54.7
AITL1_T	FF-T	26631572	3.995	2.7	3.851	96.4	3.364	87.4	85.9	62.0	54.9
AITL2_T	FF-T	23743221	3.561	2.6	3.447	96.8	3.038	88.1	88.8	63.0	57.8
AITL3_T	FF-T	23983023	3.597	2.7	3.479	96.7	3.070	88.2	88.6	62.1	55.9
AITL4_T	FF-T	24908208	3.736	2.6	3.617	96.8	3.199	88.4	88.5	62.2	55.6
AITL5_T	FF-T	24089847	3.613	2.6	3.495	96.7	3.082	88.2	88.8	63.5	57.3
AITL6_T	FF-T	25250231	3.788	2.6	3.666	96.8	3.232	88.2	89.6	67.0	59.0
AITL7_T	FF-T	22864439	3.430	2.5	3.324	96.9	2.927	88.1	88.1	60.1	56.3
AITL8_T	FF-T	30582453	4.587	2.5	4.443	96.8	3.965	89.2	91.1	71.8	57.0
AITL9_T	FF-T	30768493	4.615	2.7	4.462	96.7	4.012	89.9	90.9	71.4	54.8
Mean		25754116	3.863	2.6	3.737	96.7	3.305	88.4	89.0	64.8	56.3
SD		2637551	0.396	0.1	0.382	0.1	0.360	0.7	1.4	3.8	1.4

Abbreviations: FFPE-T: formalin-fixed paraffin-embedded tissues; FF-T: fresh frozen tissue.

Supplementary Table 2: Details of shortlisted pathogenic mutations identified by WES

Sample ID	Sample ID	Gene	CDS*	Protein	AAF	Somatic status*
AITL-1	AITL-1	TET2	c.5271delT	p.S1758fs*5	0.13	Somatic
	AITL-1	TET2	c.4553C>G	p.S1518*	0.17	Somatic
AITL-2	AITL-2	TET2	c.3320_3321insA	p.P1108fs*22	0.15	Somatic
	AITL-2	TET2	c.4519C>T	p.Q1507*	0.31	reported in Cosmic
AITL-3	AITL-3	TET2	c.1802delC	p.S602fs*37	0.08	reported in Cosmic
AITL-4	AITL-4	TET2	c.2021_2028delAG TCACTG	p.Q674fs*4	0.11	Somatic
	AITL-4	TET2	c.3811_3812insG	p.C1271fs*29	0.21	reported in Cosmic
AITL-5	AITL-5	TET2	c.4932_4945delATAT CTGGGTTCCT	p.Y1645fs*11	0.17	n/a
	AITL-5	TET2	c.5469_5470insATTA	p.G1824fs*23	0.28	n/a
AITL-6	AITL-6	TET2	c.5636A>G	p.E1879G	0.37	Somatic
AITL-7	AITL-7	TET2	c.2012delC	p.A671fs*29	0.07	Somatic
	AITL-7	TET2	c.4707C>A	p.Y1569*	0.14	reported in Cosmic
AITL-8	AITL-8	TET2	c.3061C>T	p.Q1021*	0.13	reported in Cosmic
AITL-9	AITL-9	TET2	c.2247_2253delAA TAAAG	p.N752fs*59	0.15	Somatic
	AITL-9	TET2	c.3873G>A	p.W1291*	0.19	Somatic
AITL-1	AITL-1	DNMT3A	c.915G>A	p.W305*	0.17	Somatic
AITL-4	AITL-4	DNMT3A	c.2381_2382insT	p.W795fs*3	0.33	Somatic
AITL-5	AITL-5	DNMT3A	c.2645G>A	p.R882H	0.21	reported in Cosmic
	AITL-5	IDH2	c.516G>C	p.R172S	0.14	reported in Cosmic
AITL-6	AITL-6	IDH2	c.514A>G	p.R172G	0.21	reported in Cosmic
AITL-8	AITL-8	IDH2	c.515G>A	p.R172K	0.13	reported in Cosmic
AITL-2	AITL-2	RHOA	c.50G>T	p.G17V	0.14	reported in Cosmic
AITL-3	AITL-3	RHOA	c.50G>T	p.G17V	0.23	reported in Cosmic
AITL-6	AITL-6	RHOA	c.50G>T	p.G17V	0.24	reported in Cosmic
AITL-3	AITL-3	PLCG1	c.2606G>A	p.G869E	0.14	Somatic
AITL-6	AITL-6	PLCG1	c.1034C>T	p.S345F	0.22	reported in Cosmic
AITL-1	AITL-1	TNFRSF21	c.1282delA	p.S428fs*51	0.18	Somatic
AITL-2	AITL-2	CCND3	c.838C>T	p.Q280*	0.24	Somatic
AITL-8	AITL-8	SAMSN1	c.457C>T	p.R153*	0.61	reported in Cosmic

*All variants included in this table are confirmed by Sanger sequencing. In cases 1, 2, 4, 6, 7 and 9 in which germline DNA was available, Sanger sequencing of the germline DNA was carried out to confirm whether the variant identified is somatic.
n/a: data not available.

Supplementary Table 3: Summary of AITL cases investigated by whole exome sequencing

Hospital	Case number	Nature of tissue specimen	Primary site of Lymphoma	Age	Sex	Immunophenotype of the neoplastic T-cells
UK	AITL-1	Frozen cell suspension	lymph node	75	M	CD3+, CD4+, Prominent extrafollicular expansion of FDC meshworks as shown by CD21 staining, and florid proliferation of high endothelial venules
UK	AITL-2	Frozen tissue (tumour confirmed by HE)	lymph node	71	M	CD3+, CD5+, CD10+, Prominent extrafollicular expansion of FDC meshworks as shown by CD23 staining, and florid proliferation of high endothelial venules
UK	AITL-3	Frozen tissue (tumour confirmed by HE)	lymph node	65	M	CD3+,CD4+,CD5+, CD10+ Prominent extrafollicular expansion of FDC meshworks
Taiwan	AITL-4	Frozen tissue (tumour confirmed by HE)	lymph node	65	M	CD3+,CD4+, CD10+, BCL6−, PD-1+ Prominent expansion of FDC meshworks and prominent proliferation of high endothelial venules
Taiwan	AITL-5	Frozen tissue (tumour confirmed by HE)	lymph node	57	F	CD3+, CD10+, BCL6−, PD-1− Prominent expansion of FDC meshworks and moderate proliferation of high endothelial venules
Taiwan	AITL-6	Frozen tissue (tumour confirmed by HE)	lymph node	80	F	CD3+, CD10+, BCL6−, PD-1+ Prominent expansion of FDC meshworks and florid proliferation of high endothelial venules
Taiwan	AITL-7	Frozen tissue (tumour confirmed by HE)	lymph node	82	F	CD3+, CD4+, CD10+, BCL6+, PD-1+ Prominent extrafollicular expansion of FDC meshworks by CD21 staining
Taiwan	AITL-8	Frozen tissue (tumour confirmed by HE)	lymph node	63	M	CD3+, CD4+, CD10−, BCL6−, PD-1+ Prominent expansion of FDC meshworks by CD21 staining and florid proliferation of high endothelial venules
Taiwan	AITL-9	Frozen tissue (tumour confirmed by HE)	lymph node	63	F	CD3+, CD4+, CD10+, BCL6+, PD-1+ Prominent extrafollicular expansion of FDC meshworks by CD21 staining and florid proliferation of high endothelial venules

Abbreviation: FDC: follicular dendritic cell.
